# Host and parasite identity interact in scale-dependent fashion to determine parasite community structure

**DOI:** 10.1007/s00442-023-05499-3

**Published:** 2024-01-11

**Authors:** Joshua I. Brian, David C. Aldridge

**Affiliations:** 1 Aquatic Ecology Group, Department of Zoology, University of Cambridge, The David Attenborough Building, Cambridge, CB2 3QZ UK; 2 Present Address: Department of Geography, Bush House North East, King’s College London, London, WC2B 4BG UK

**Keywords:** Community assembly, Coinfection, Dispersal, Ecological scale, Metacercariae

## Abstract

Understanding the ecological assembly of parasite communities is critical to characterise how changing host and environmental landscapes will alter infection dynamics and outcomes. However, studies frequently assume that (a) closely related parasite species or those with identical life-history strategies are functionally equivalent, and (b) the same factors will drive infection dynamics for a single parasite across multiple host species, oversimplifying community assembly patterns. Here, we challenge these two assumptions using a naturally occurring host–parasite system, with the mussel *Anodonta anatina* infected by the digenean trematode *Echinoparyphium recurvatum*, and the snail *Viviparus viviparus* infected by both *E. recurvatum* and *Echinostoma* sp. By analysing the impact of temporal parasite dispersal, host species and size, and the impact of coinfection (moving from broader environmental factors to within-host dynamics), we show that neither assumption holds true, but at different ecological scales. The assumption that closely related parasites can be functionally grouped is challenged when considering dispersal to the host (i.e. larger scales), while the assumption that the same factors will drive infection dynamics for a single parasite across multiple host species is challenged when considering within-host interspecific competition (i.e. smaller scales). Our results demonstrate that host identity, parasite identity and ecological scale require simultaneous consideration in studies of parasite community composition and transmission.

**Supplementary Information:**

The online version contains supplementary material available at 10.1007/s00442-023-05499-3.

## Introduction

Understanding drivers of community assembly and maintenance is a key goal of ecology (Vellend 2010). As individual organisms can constitute a community of parasitic taxa (Pedersen and Fenton 2007), parasites provide an ideal system for studying community assembly and structure (e.g. Mihaljevic et al. 2018; Dallas et al. 2019; Moss et al. 2020; Nielsen et al. 2020; Brian and Aldridge 2021a). Further, understanding the construction of parasite communities is important for a number of reasons: parasites are a key component of food webs (Lafferty et al. 2008), they are one of the most dominant contributors to the biomass of aquatic ecosystems (Preston et al. 2013, 2020), and their life-history dynamics have significant implications for animal and human health (Sokolow et al. 2016; Daversa et al. 2021). Further, parasites influence the structure of their host communities, which may be especially important if they infect organisms with ecosystem engineering capabilities (Friesen et al. 2020; Brian et al. 2022). Given individual hosts represent discrete and highly replicated infracommunities, a greater focus on parasite community construction has the potential to not only shed light on host-parasite interactions but also inform the ecology of free-living communities (Dallas and Presley 2014). Despite recent work in this area (e.g. Devevey et al. 2015; Rynkiewicz et al. 2019; Brian and Aldridge 2023), our understanding of parasite communities lags behind that of free-living communities (Budischak et al. 2016).

As many parasites display complex multi-host life cycles (Runghen et al. 2021), studying parasite community structure can be challenging. This has led to two implicit simplifying assumptions in the parasite community literature. The first is that closely related parasites, or those with the same life-history strategy, can be grouped into functional or phylogenetic categories for consideration and analysis, a suggestion that appears in many reviews (Johnson et al. 2015; Rynkiewicz et al. 2015; Fountain-Jones et al. 2018). For example, a recent study examined trait shifts in bovine parasite communities in response to coinfection with a microparasite, with parasites placed in coarse categories such as whether they possessed single vs. multi-host life cycles (Beechler et al. 2019). Recent meta-analyses investigating the role of parasites in host-level interactions also employ similar categories (e.g. Hasik et al. 2023). While such studies seek to identify emergent taxa- or trait-based patterns that emerge in spite of underlying species- or host-specific differences, the implication is that these will be usefully predictive for individual parasite species: based on certain characteristics of a parasite, we should be able to predict how it will respond to certain drivers, providing a useful shortcut to understand parasite communities. However, empirical evidence supporting this underlying assumption remains ambiguous. The few studies that address this question suggest that even very closely related parasites may be affected by different factors in a single host species (e.g. Williamson et al. 2019; Billet et al. 2020). Recent simulations also suggest that coarse functional groupings may oversimplify community assembly trends (Kohli and Jarzyna 2021). However, explicit consideration of this assumption remains rare in parasite studies.

The second assumption is that the responses of a single parasite species are consistent across host species for a given factor. A recent example of this assumption comes from Brian and Aldridge (2023), who studied the response of parasite communities to host- and environment-level factors across two different host mussel species, using joint species distribution models. This approach detected the influence of host size on parasite communities (generally, bigger hosts have more parasites), but could not directly account for the fact that the importance of host size may vary with the different host species. Joint species distribution models and similar approaches which average across different host species are becoming increasingly common in parasitology (e.g. Dallas et al. 2019; Sallinen et al. 2020; Brian and Aldridge 2021a). However, each host species (and indeed each host individual) represents a discrete environment, the colonisation of which may rely on a unique set of processes (Johnson et al. 2015; Rynkiewicz et al. 2015). Host physiology has an important role to play in determining responses to infection, leading different host species to potentially experience higher prevalences or intensities of a certain parasite than others (Albery and Becker 2021). This is particularly relevant as it is likely that many actual host-parasite associations have not been observed (Dallas et al. 2017), and so conclusions about host-parasite relationships are drawn from only common hosts (Dallas et al. 2020). Without knowing how a parasite responds to all its hosts, ecologically relevant hypotheses such as the dilution effect (Ostfeld and Keesing 2012) remain difficult to appropriately assess. This will be especially true for invertebrate hosts which have been significantly understudied (Wilson et al. 2015), yet act as intermediate hosts for many heteroxenous parasites (Schwelm et al. 2020).

Both these assumptions regarding infection dynamics (closely related parasite species have functionally equivalent dynamics; the same factors govern a single species occurring in multiple hosts) have the potential to oversimplify conclusions concerning parasite community assembly, yet have received little critical consideration. In particular, it is unclear how these assumptions are influenced by ecological scale. The scale of study (e.g. between-site, through time, between-host or within-host) is a key consideration when determining the main factors responsible for community assembly and maintenance (Penczykowski et al. 2016; Bolnick et al. 2020a; Rynkiewicz et al. 2019; Moss et al. 2020). The interplay between scales is also important (Johnson et al. 2015): factors may be important at one scale but not others (Bolnick et al. 2020b), and processes operating at a higher scale may mask those at a lower scale if not properly accounted for (Brian and Aldridge 2023). As such, the two assumptions may be challenged at different scales. For example, the same parasite species may preferentially target larger hosts of multiple species equally (e.g. Brian and Aldridge 2023), but may then be facilitated by a coinfecting parasite in one species and impeded by a coinfector in another, altering community-level transmission (e.g. Mordecai et al. 2016). If only regional or between-host scales were considered (without taking into account the within-host environment), the natural conclusion would be that the parasite preferentially targets the first host. Alternatively, two related parasite species may have different temporal or spatial infection dynamics, but react in similar fashion when challenged with a coinfecting parasite. For example, parasites of small mammals vary seasonally and with site, but functionally similar parasite species respond to coinfection in similar ways (Dallas et al. 2019). If sampling occurred at a single site or time period, the two species may be incorrectly classed as functionally equivalent.

**Fig. 1:**
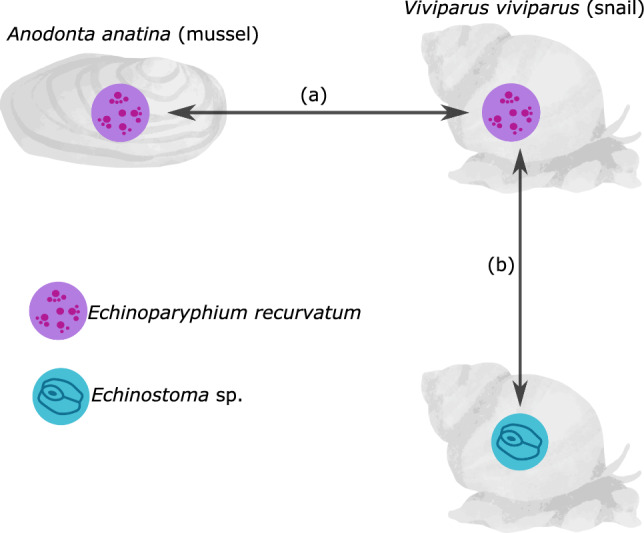
The triangulated host–parasite study system, where *V. viviparus* hosts both metacercariae, while *A. anatina* hosts only *E. recurvatum*. **a** Comparing infection dynamics of *E. recurvatum* between two host species challenges the assumption that the same factors explain infection dynamics across multiple host species for a single parasite. **b** Comparing infection dynamics between *E. recurvatum* and *Echinostoma* sp. challenges the assumption that species with identical life-history strategies are functionally equivalent

The scope of these assumptions and their scale-dependency will vary from system to system. However, these issues will be particularly relevant for guilds of generalist, closely related parasites that appear equivalent in terms of their life-history. We take advantage of one such group, digenean trematode metacercariae which show low host specificity (Johnson et al. 2020), to challenge the two assumptions we have highlighted and show that accurate characterisation of parasite communities requires nuanced life-history information across multiple scales. Specifically, we study two closely related metacercariae populations across sympatric populations of two host mollusc species (Fig. 1), the duck mussel *Anodonta anatina* (that hosts *Echinoparyphium recurvatum*) and the common river snail *Viviparus viviparus* (that hosts both *E*. *recurvatum* and *Echinostoma* sp.). This system allows for a direct assessment of two related questions (Fig. 1): (a) how does the same parasite respond to abiotic and biotic drivers in different host species (comparing the responses of *E. recurvatum* in *A. anatina* and *V. viviparus*, Fig. 1a); and (b) do two parasites with comparable life histories respond in the same fashion to these drivers in a single host species (comparing the responses of *E. recurvatum* and *Echinostoma* sp. in *V. viviparus*, Fig. 1b). By taking into account the effect of seasonality, host size and coinfection with castrating digenean trematodes (‘castrators’), we analyse the effect of scale on these conclusions (Table 1). We also examine both prevalence and intensity of infection, as these can be influenced by different factors (Brian and Aldridge 2021b). We show that a single parasite species varies in its response to castrators between host species, but seasonal patterns and the influence of host size are consistent regardless of host species. In contrast, the two different parasite species show different seasonality and responses to host size, but consistent responses to castrators within the host. Our conclusions highlight the nuances required to accurately understand parasite community construction, but also that a consideration of scale may be useful in predicting when simplifying assumptions can and cannot be made.

**Table 1:** Assumptions tested in the paper, with accompanying hypotheses

		Large scale $\to$ small scale		
Assumption	Comparison	Seasonality	Host size	Response to coinfecting castrators
Assumption 1: The same factors explain dynamics of a single parasite species across multiple host species	*E. recurvatum* in mussels *vs*. *E. recurvatum* in snails (Fig. 1a)	H1: *E. recurvatum* shows same patterns of seasonality in both host species	H3: *E. recurvatum* responds to changes in mussel and snail size in the same way	H5: *E. recurvatum* interacts in the same way with coinfecting castrators in both snails and mussels
Assumption 2: Two parasite species with identical life-history strategies are functionally equivalent	*E. recurvatum* in snails *vs*. *Echinostoma* sp. in snails (Fig. 1b)	H2: *E. recurvatum* and *Echinostoma* sp. show the same patterns of seasonality	H4: *E. recurvatum* and *Echinostoma* sp. respond in the same way to changes in host size	H6: *E. recurvatum* and *Echinostoma* sp. respond in the same way to the presence of castrators

## Methods

### Sampling procedure and dissection

We sampled two host species: the unionid mussel *Anodonta anatina* (henceforth “mussels”) and the prosobranch snail *Viviparus viviparus* (“snails”) (Fig. 1). Both species act as second intermediate hosts for digenean trematodes, which occur in the gonads (*A. anatina*) or gonads and pericardial sac (*V. viviparus*) as metacercariae (Brian and Aldridge 2019). *A. anatina* hosts *Echinoparyphium recurvatum* (identified following Brian and Aldridge 2021a), while *V. viviparus* hosts both *E. recurvatum* and *Echinostoma* sp. (identified to genus only given difficulty in assigning species to this genus; Stanicka et al. 2020). Additional sources consulted for identification were Kanev (1994), Chai et al. (2011), and Zhytova et al. (2019). We sampled mussels and snails from the Old West River at Stretham (52.3343° N, 0.2243° E), a tributary of the River Great Ouse (UK), at monthly time points from February 2019 to February 2020. At each sampling point, 60 mussels and between 30 and 44 snails were collected, yielding a total sampling effort of 720 mussels and 374 snails. The molluscs were stored in 10 L buckets of river water at 8 °C in the laboratory until dissection; all dissections were carried out within 72 h of collection.

In mussels, metacercariae prevalence and intensity were quantified by isolating the visceral mass of the mussel and inspecting replicate squashes of gonad tissue (the major site of infection of *E. recurvatum*) under light microscopy, following Brian and Aldridge (2020). Since metacercariae can be found in multiple tissues in snails, all tissue was squashed and inspected as above to identify and count metacercariae in this host. In both host species, these squashes also identified infection with castrating trematodes which use the molluscs as first intermediate hosts: *Rhipidocotyle campanula* in mussels, and an unidentified Himasthlidae (following Hechinger et al. 2011) in snails (hereafter collectively referred to as ‘castrators’).

### Statistical analysis

Broadly, we explored drivers of metacercariae presence and intensity across three different scales (see full hypotheses in Table 1). At the largest scale, the total number of parasite infective stages in the environment was captured by the month of sampling (i.e. seasonality), if we assume that a larger infective pool leads to greater prevalences and/or intensities of infection (D’Bastiani et al. 2020). Within each month at an intermediate scale, we expect there to be variation in the hosts successfully colonised by parasites (Guégan et al. 2005); we evaluated this by modelling the effect of both host identity (species) and host size (length, in mm; see Table S1). At the smallest scale, parasite success, in terms of both persistence or abundance, can be modified by interspecific interactions with coinfecting taxa (Knowles et al. 2013; Vaumourin et al. 2015); we examined the impact of coinfection by modelling the influence of castrator presence (*R. campanula* in mussels and Himasthlidae in snails) on the two metacercariae species of interest. All data analysis and visualisation were executed with R v.3.6.3 (R Core Team 2020).

We first confirmed that *E. recurvatum* and *Echinostoma* sp. occurred independently in snails, the host where they both occur (*χ*^2^_1_ = 3.49, *p* = 0.06), and thus we modelled the two species separately. As we had no a priori hypotheses for how our measured variables, or their interactions, would affect metacercariae, we took an exploratory approach and began with full models including all possible explanatory variables (month, host species, host length, presence of castrators) and their interactions. The *Echinostoma* sp. models did not have host species terms, given they were only found in snails. Separate models were constructed for metacercariae prevalence, and metacercariae intensity (which only included infected hosts, following Bush et al. 1997). Intensity was recorded as the total number of metacercariae observed *per* host. Prevalence models used logistic regression, while intensity models used negative binomial regression (MASS package, Venables and Ripley 2002) given extensive overdispersion present in the count data. Both sets of models were confirmed to fit the data well (see supplementary code). In total, we were left with four full models, describing *E. recurvatum* prevalence, *E. recurvatum* intensity, *Echinostoma* sp. prevalence and *Echinostoma* sp. intensity.

From each full model, we assessed all possible sub-models, and selected the combination of variables which best described the data, using AICc and the dredge function of the MuMIn package (Barton 2020), where ΔAICc was < 2 between the best model and other models, we used parsimony to select the model with the fewest parameters (Burnham and Anderson 2004). We then refitted the selected model to obtain final parameter estimates for each variable included in the chosen model. We used ggplot2 (Wickham 2016) to visualise results. For the purposes of presentation in the Results, *p* values denote goodness-of-fit tests comparing models with and without the variable of interest. Full model results including β-parameters, standard errors and *p* values for levels of each categorical variable can be found in Tables S2 (*E. recurvatum*) and S3 (*Echinostoma* sp.).

## Results

In this study, we modelled the impact of seasonality (using sampling month as a proxy), host size (length in mm) and coinfection with castrating trematodes (‘castrators’) on the prevalence and intensity of two types of metacercariae, across two different host species. This approach enabled us to not only ascertain the importance of factors at different scales on parasite population trends, but also how the influence of those factors varies across different hosts and across related parasite species. Summaries of the model outputs are presented below: for full model outputs, see Table S2 (*E. recurvatum*) and Table S3 (*Echinostoma* sp.). Broad results corresponding to the hypotheses in Table 1 can be found in Table 2.

**Table 2:** Results of all hypotheses (see Table 1)

		Large scale $\to$ small scale		
Assumption	Comparison	Seasonality	Host size	Response to coinfecting castrators
Assumption 1: The same factors explain dynamics of a single parasite species across multiple host species	*E. recurvatum* in mussels *vs*. *E. recurvatum* in snails (Fig. 1a)	H1: SUPPORTED for prevalence (Fig. 2a), NOT SUPPORTED for intensity (Fig. 2b)	H3: SUPPORTED for prevalence (Fig. 3a vs. Fig. 3b) and intensity (Fig. 3c vs. Fig. 3d)	H5: NOT SUPPORTED for either prevalence (Fig. 3e) or intensity (Fig. 3f)
Assumption 2: Two parasite species with identical life-history strategies are functionally equivalent	*E. recurvatum* in snails *vs*. *Echinostoma* sp. in snails (Fig. 1b)	H2: NOT SUPPORTED for either prevalence (Fig. 2a vs. Fig. 2b) or intensity (due to randomness of *Echinostoma* sp. intensity)	H4: NOT SUPPORTED for either prevalence (Fig. 3b vs. Fig. 4a) or intensity (due to randomness of *Echinostoma* sp. intensity)	H6: SUPPORTED for prevalence (Fig. 3e vs. Fig. 4b/4c), NOT SUPPORTED for intensity (due to randomness of *Echinostoma* sp. intensity)

Molluscs across the full size range of both hosts were sampled (mussels 28–89 mm, 63.9 ± 11.1 mm [mean ± s.d.]; snails 5–14.5 mm, 9.7 ± 1.4). Average sizes were consistent for both species throughout the year (Table S1).

### Patterns of seasonality (hypotheses 1 and 2)

**Fig. 2:**
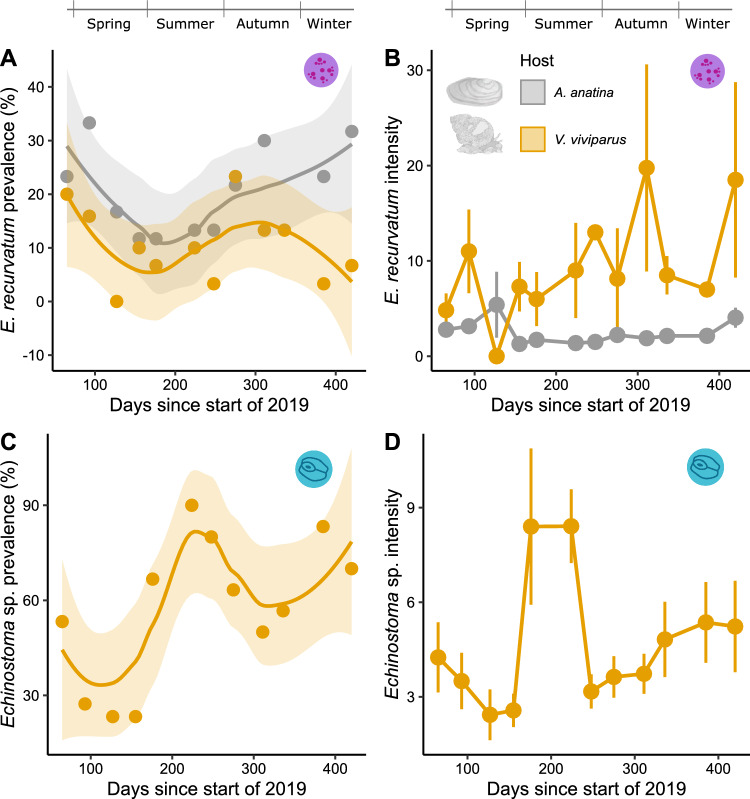
Overall prevalence and intensity trends for *E. recurvatum* (purple metacercariae) and *Echinostoma* sp. (blue metacercariae) in both their mussel host *A. anatina* (grey) and snail host *V. viviparus* (yellow) (*N* = 720 mussels, 374 snails). **A** Prevalence of *E. recurvatum* through the sampling period. Dots represent raw prevalence values in each host species, with lines representing a loess-estimated smoothing function, and shading representing a 95% confidence interval. **B** *E. recurvatum* intensities across host species through the sampling period (± 1 standard error). **C** Prevalence of *Echinostoma* sp. through the sampling period. Details as per graph (**A**). **D** *Echinostoma* sp. intensity through the sampling period (± 1 standard error)

Seasonality was an important factor governing the prevalence of *E. recurvatum*, which varied throughout the year (*p* < 0.001). In support of hypothesis 1, this trend was consistent between the two host species, shown by a lack of interaction between host and time (*p* = 0.231; Fig. 2A), but prevalences were consistently 5–10% higher for the mussels than snails (*p* < 0.001; Fig. 2A). However, not supporting hypothesis 1, *E. recurvatum* infection intensity showed the opposite trend, with no temporal pattern (*p* = 0.906), but infections that were 3.7 times more intense on average in snails than mussels (*p* = 0.008, Fig. 2B). This difference between hosts was also dependent on host length and the presence of castrators (explored further below).

The prevalence of *Echinostoma* sp. was also significantly time-dependent (*p* < 0.001) but in a different fashion to *E. recurvatum*, with a peak in late summer as opposed to a depression (Fig. 2C), and a corresponding peak in intensity (Fig. 2D). Therefore, hypothesis 2 was not supported.

### Effect of host characteristics and coinfection with castrators on *E. recurvatum* (hypotheses 3 and 5)

**Fig. 3:**
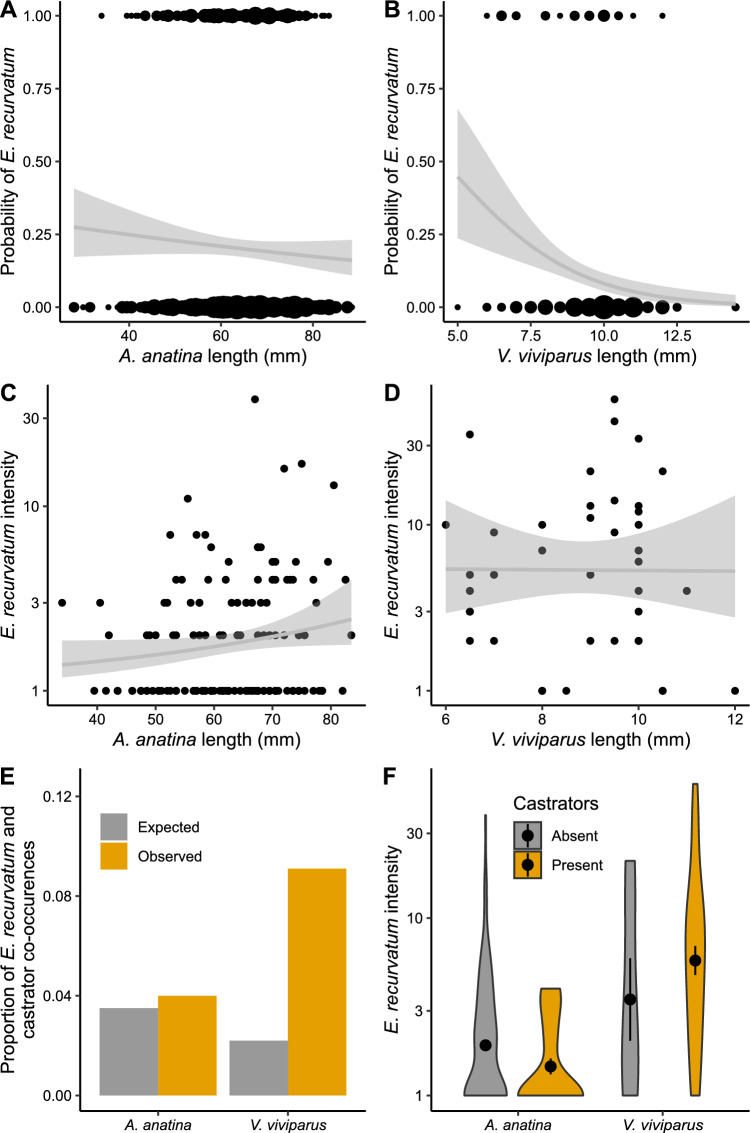
Factors influencing *E. recurvatum* prevalence and intensity in their mussel (*A. anatina*) and snail (*V. viviparus*) hosts (*N* = 720 mussels, 374 snails). **A** Increasing host length reduces the odds of infection in both mussels, and (**B**) snails. Size of points is proportional to the number of observations. **C** Host length increases infection intensity in mussels, but has little effect (**D**) in snails. Note log-transformed y-axis in both cases. For (**A**–**D**), lines represent predictions from final fitted models, with shading corresponding to a 95% confidence interval. **E** Coinfection with castrators has host-specific impacts on both *E. recurvatum* prevalence and (**F**) intensity. Black dots and error bars represent mean infection intensity ± 1 standard error

The effect of host length was consistent among host species and was an important predictor of both prevalence and intensity, but in opposite fashion. For both mussels (Fig. 3A) and snails (Fig. 3B), increased host length led to reduced likelihood of infection (*p* = 0.023), supporting hypothesis 3. However, in those hosts that were infected, an increased size was correlated with higher infection intensities (*p* = 0.006), though this effect was mainly observed in mussels (Fig. 3C). There was high variability observed in the infection intensities of snails (Fig. 3D), though statistically the effect of length was consistent among hosts (lack of a host:length interaction, *p* = 0.392), also supporting hypothesis 3.

Prevalence and intensity of *E. recurvatum* infection was dependent on coinfection with castrators, an effect that varied with host identity (host:castrator interaction: *p* < 0.001 [prevalence]; *p* = 0.039 [intensity]); therefore, hypothesis 5 was not supported. In their mussel hosts, *E. recurvatum* and their castrators co-occurred in proportion with their respective prevalences; however, in their snail hosts, they co-occurred far more frequently than expected by chance (Fig. 3E). Similarly, the intensity of *E. recurvatum* was 1.7 times lower when coinfecting with castrators in mussels, but 1.5 times higher when coinfecting with castrators in snails (Fig. 3F).

### Effect of host characteristics and coinfection with castrators on *Echinostoma* sp. and comparison with *E. recurvatum* (hypotheses 4 and 6)

There was a significant interaction between time and host length explaining infection intensity (*p* < 0.001); this was shown to be caused by highly variable infection intensities across months (Fig. S1), with no other factors explaining intensity of *Echinostoma* sp. Host lengths did not vary significantly through time (Table S1), and so cannot explain this pattern. It appears *Echinostoma* sp. intensity is either explained by additional unmeasured factors, or is largely stochastic.

**Fig. 4:**
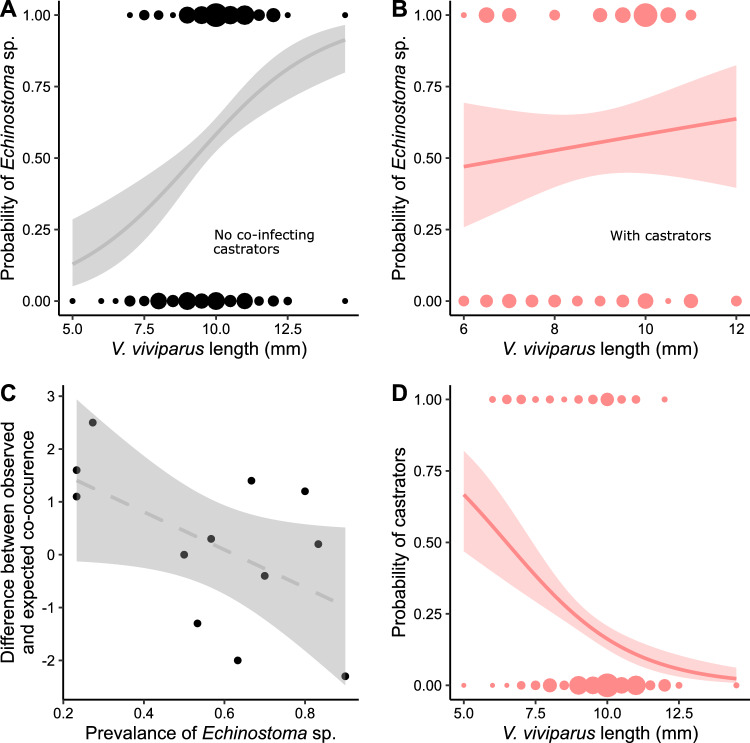
Factors influencing the prevalence of *Echinostoma* sp. in its snail host *V. viviparus*. For all four panels, shading corresponds to a 95% confidence interval, and size of dots is proportional to the number of observations. **A** Increasing host length increased probability of infection in hosts without castrators (grey points and line, *N* = 298), but not for **B** hosts where castrators were present (red points and line, *N* = 76). Lines represent predictions from final fitted models. **C** Relationship between *Echinostoma* sp. prevalence and the difference between observed and expected (i.e. O minus E) number of co-occurrences with castrators. Dashed line indicates limited statistical support for the relationship (*N* = 12 sampling months). **D** Increasing host length was associated with a much lower probability of infection with castrators (red points and line) (*N* = 374). Line is fitted with a GLM (logistic link)

*Echinostoma* sp. prevalence was governed by a complex set of interactions between host length, month and presence of castrators. First, the effect of host length was dependent on the presence of castrators (length:castrator interaction, *p* = 0.033). In the absence of castrators, increased length increased the probability of infection (Fig. 4A), opposite to *E. recurvatum* (not supporting hypothesis 4). However, this effect largely disappeared in the presence of castrators (Fig. 4B). There was also a castrator:month interaction (i.e. the relationship between castrators and *Echinostoma* sp. varied between months, *p* = 0.007). However, both interactions can be at least partially explained by considering *Echinostoma* sp. and castrator population dynamics. Regarding the castrator:month interaction, there was a moderate relationship between the observed co-occurrences and *Echinostoma* sp. prevalence (*F*_1, 10_ = 4.44, *p* = 0.061, Fig. 4C): at low *Echinostoma* sp. prevalences, it appeared to co-occur with castrators more than expected by chance, while at high prevalence it occurred less than expected by chance. Regarding the length:castrator interaction (Fig. 4A, B), we further noted that castrators disproportionately occurred in smaller hosts (*χ*^1^ = 26.1, *p* < 0.001, Fig. 4D), and it is in these smaller hosts where the length:castrator interaction was most strongly observed (note that *Echinostoma* sp. infection probability is ~ 10% in small hosts in the absence of castrators, Fig. 4A, but is ~ 50% in small hosts in the presence of castrators, Fig. 4B). Therefore, infection with castrators appears to facilitate *Echinostoma* sp. prevalence (just like *E. recurvatum,* supporting hypothesis 6), especially when *Echinostoma* sp. is less prevalent and hosts are small.

## Discussion

Parasites play a central role in the structure of their host communities (Dairain et al. 2020; Friesen et al. 2020) and in wider ecosystem stability and connectivity (Lafferty et al. 2008; Runghen et al. 2021). Knowledge of ubiquitous rules that contribute to parasite community assembly, and the scale at which they operate, remains largely absent (Poulin 2019), despite evidence accumulating from an increasing list of hosts. Our triangulated study system (Fig. 1) allowed us to explore two major complexities in parasite community ecology. Importantly, we have highlighted that closely related, ‘functionally equivalent’ parasites follow different infection trajectories, and that the same parasite species responds to coinfection in different ways across host species. The scale-dependency of these contrasts (Table 2) necessitates a careful consideration of the level at which parasite community composition is considered, as well as acknowledgement of the potential for different host-parasite combinations to follow trajectories that cannot be predicted by host or parasite identity alone.

### Infection dynamics of the same parasite species is moderated by variable within-host interspecific interactions

**Fig. 5:**
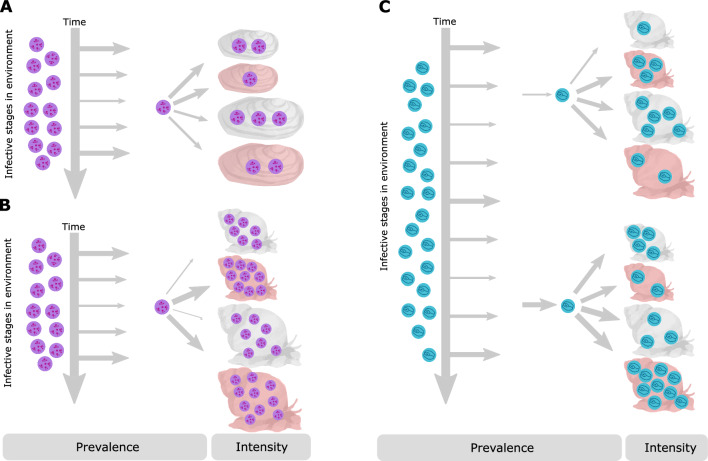
Summary of infection dynamics through time of *E. recurvatum* (purple metacercariae) and *Echinostoma* sp. (blue metacercariae), in small and large hosts, some of which are also infected with castrators (red hosts). The time scale is ~ 1 year for all three panels. Arrow widths are proportional to strength of effect and directly comparable across all panels; indications of intensity are similarly reflective of mean infection intensity in each case. **A**, **B** Infection dynamics for *E. recurvatum* in mussels *A. anatina* (**A**) and snails *V. viviparus* (**B**). Prevalence changes through the year, likely reflecting propagule pressure. Smaller hosts are more likely to be infected, but this effect is outweighed by the positive influence of castrators in *V. viviparus*, which do not affect prevalence in *A. anatina*. In contrast, intensity increases with larger hosts, and also increases with castrator presence in *V. viviparus* but decreases with castrator presence in *A. anatina*. **C** Infection dynamics for *Echinostoma* sp. Propagule pressure varies throughout the year, in different fashion to A and B. Likelihood of infection increases with larger hosts, and also with castrator presence, but only when propagule pressure is low and disproportionately in small hosts (top half of panel **C**). Intensity of infection is highly variable, and unrelated to propagule pressure, host size or castrator presence

The parasite *E. recurvatum* occurs in both mussels and snails, and shows similar patterns of prevalence through time (Fig. 2A). This result is reassuring and intuitive, as it suggests that prevalence depends on seasonal variation in propagule pressure from the environment: as both hosts occur in the same site and experience the same conditions, propagule pressure should be the same for both hosts (Fig. 5A, B). The greater overall prevalence in mussels (Fig. 2A) is likely explained by the high filtering capacity of unionid mussels (Tankersley and Dimock 1993) combined with their larger average size than snails. Despite the large difference in size between host species, the overall effect of size was also consistent across hosts: larger hosts generally had a lower likelihood of hosting *E. recurvatum*, but a higher intensity when they did (Fig. 5A, B). The mechanism for this pattern cannot conclusively be established in an observational context; we suggest that *E. recurvatum* increases host mortality, either directly through nutrient absorption (e.g. Mischler et al. 2016) or indirectly through increasing the likelihood of predation (e.g. Addino et al. 2010), and host individuals are thus more likely to reach larger sizes if uninfected. However, in those individuals that are infected and survive, a greater lifetime exposure would lead to the observed higher intensities. In other words, patterns of prevalence and intensity may be due to host age, which is typically reflected in host size in bivalves (Ollard and Aldridge 2023). In sum, broad temporal and host characteristics are consistent for this parasite species, regardless of host species identity.

However, coinfection with castrating trematodes altered both prevalence and intensity in host-specific fashion. In mussels, castrators had little effect on *E. recurvatum* prevalence but reduced intensity (Fig. 5A), while castrators in snails significantly increased both prevalence and intensity (Fig. 5B). We interpret castrators as facilitating or inhibiting metacercariae, rather than the other way around, given metacercariae show little competitive ability (Poulin 2001, but see Billet et al. 2020). It is likely that castrators in snails weaken the immune system or alter host behaviour, making successful infection by metacercariae more likely (Magalhães et al. 2015; Fig. 5B). It has been shown that infection with castrators significantly affects energy budgets of molluscs, and thus their ability to cope with other stressors (Jokela et al. 2005; Lafferty and Kuris 2009). In contrast, any immune response by mussels to castrator infection does not appear to cascade to facilitating *E. recurvatum* (Fig. 5A). Intensity differences can similarly be explained by within-host dynamics: *E. recurvatum* only infects the gonad of mussels, where it is in direct competition with castrators and, therefore, occurs in lower intensities in the presence of coinfection (Fig. 5A). This aligns with previous research that there are more negative interactions between parasites occupying the same host tissue (Knowles et al. 2013; Griffiths et al. 2014; Henrichs et al. 2016; Dallas et al. 2019; Brian and Aldridge 2021a). In contrast, *E. recurvatum* occupies other tissues of snails in addition to the gonad, and is, therefore, released from spatially driven interspecific competition, and can thus attain higher intensities (Fig. 5B). Therefore, despite having identical infection dynamics in terms of dispersing to hosts, the host species quantitatively differ in their likelihood of transmitting infection to definitive hosts due to within-host coinfection dynamics. As metacercarial transmission to the final host occurs via consumption, these quantitative differences are likely to be reflected in the parasite burdens of the final hosts.

### Infection dynamics of closely related parasite species are moderated by temporal context and host characteristics

Snails host both *E. recurvatum* and *Echinostoma* sp., providing an opportunity to compare infection trajectories of two ‘functionally equivalent’ species. *Echinostoma* sp. appears to be much more host specific, with an average prevalence much higher than *E. recurvatum* (Fig. 2A, C), and no evidence of infection in mussels despite mussels being extensive filter-feeders and attaining higher biomass in the system. This host specificity shows the possible importance of co-evolutionary history in host-parasite studies (Brannelly et al. 2021), and that sympatric parasite species faced with the same host landscape may follow very different infection dynamics.

Similar to *E. recurvatum*, *Echinostoma* sp. is facilitated by castrators in terms of prevalence, potentially through castrators weakening host resources and thus making them more vulnerable to subsequent infection (Magalhães et al. 2015). This relationship is highly supported by our data, given this trend was largely observed in smaller hosts (Fig. 4B) where castrators were much more common (Fig. 4D). This trend does appear to break down in months where *Echinostoma* sp. prevalence was very high (Fig. 4C). We suggest this is due to a consistent low prevalence of castrators—when propagule pressure of *Echinostoma* sp. is extremely high, most hosts (up to 90%, Fig. 2C) will be infected anyway, and so facilitation is observed disproportionately at times of low propagule pressure (Fig. 5C). In any case, the effects of castrators are qualitatively similar between *E. recurvatum* and *Echinostoma* sp.

In contrast, the temporal pattern of prevalence differs between the two parasite species (Fig. 5B, C); hosts are under pressure from different parasite species at different times. This is supported by previous work which also shows diverse temporal infection patterns in closely related trematode species (Granovitch et al. 2000), something that is not captured if the parasites are treated as functionally equivalent. Similarly, while larger host snails were less likely to host *E. recurvatum*, they were more likely to host *Echinostoma* sp. (Fig. 5C), suggesting broad differences in infection dynamics between these two species. The contrast between these metacercariae in one host species, therefore, shows the opposite trend to the comparison of one metacercaria across host species: here, the within-host coinfection patterns are consistent, but the wider dynamics of infection are not. Our results clearly emphasise the need to better integrate host-parasite co-evolutionary dynamics into parasitology as has recently been suggested, as even very similar parasites will show different relationships to host species based on evolutionary history and ecological context (Blasco-Costa et al. 2021).

### Implications for parasite community dynamics and transmission

We have shown that the same parasite species should encounter multiple host species and respond to broad host characteristics in consistent fashion, and that host-specific differences may occur predominantly at the within-host level. In contrast, two similar parasite species show variable infection dynamics at larger scales, but respond consistently to within-host interspecific competition. Our results, therefore, challenge two common assumptions employed in parasite community ecology studies (Table 1, Fig. 1), but they also suggest that accounting for the scale-dependent nature of community construction can mitigate the impact of these assumptions (Table 2). The first assumption (closely related parasite species have functionally equivalent dynamics) is most likely to be incorrect at larger scales, while the second assumption (the same factors govern a single species occurring in multiple hosts) is most likely to be incorrect at smaller scales. While the results that different parasites exhibit differences in infection and the same parasite shows contrasting responses across host species are not inherently surprising, the different scales at which this occurs is an interesting and previously unexplored extension to parasite community assembly.

These conclusions have major implications for understanding parasite biogeography. As different host populations may vary in terms of size structure (Bolnick et al. 2020a), age distribution (Izhar and Ben-Ami 2015) or genotype composition (Sallinen et al. 2020), all of which may affect parasite communities, nuanced host-specific infection dynamics may amplify over larger scales. For example, we found that the two metacercariae occur in different prevalences across host species, and respond in opposite fashion to host size. If multiple host communities differ in the relative proportion of these two hosts, and the size distribution of each host population also varies across communities, a large proportion of variance in parasite community structure is lost by assuming the two parasites to be functionally equivalent. Further, parasites can also show intraspecific trait variation (Gervasi et al. 2015), leading to the same parasite having different average effects across populations (Reichard et al. 2015; Bolnick et al. 2020b). We cannot begin to capture this variation if parasites are treated in terms of functional or phylogenetic groups. Our study was carried out at a single site; we now recommend a similar critical analysis of the two assumptions at multiple-site or regional scales to ascertain the generality of our conclusions.

Finally, our results have implications for incorporating host-specific coinfection dynamics into studies of parasite transmission. Previous work has shown that the presence or absence of a coinfecting parasite can alter transmission of infective stages of a focal parasite to the next host, when considering a single host species (Susi et al. 2015; Sweeny et al. 2020). We extend this by showing that these effects may reverse based on host identity: mussels infected with castrators were less likely to host metacercariae that go on to infect the next host, while the opposite was true for snails. General biodiversity–disease relationships, such as the suggestion that overall community-level transmission depends on the relative presence of high- and low-competency hosts (Johnson et al. 2013; Mihaljevic et al. 2018; Halliday et al. 2020), may need to be modified to reflect the fact that a lower competency host can become much more competent depending on infection by another species.

### Supplementary Information

Below is the link to the electronic supplementary material.

Supplementary file1 (PDF 238 kb)

## Data Availability

All data supporting the manuscript can be found at https://github.com/brianjosh/parasite-assumptions

## Code availability

All code required to replicate the analyses can be found at https://github.com/brianjosh/parasite-assumptions
