# Supplementary material for: Host and parasite identity interact in scale-dependent fashion to determine parasite community structure
Source: Oecologia. 2024 Jan 11;204(1):199–211. doi: 10.1007/s00442-023-05499-3 (PMC10830602; doi:10.1007/s00442-023-05499-3)
Supplement: Supplementary file 1 — Supplementary file1 (PDF 238 kb) [file 442_2023_5499_MOESM1_ESM.pdf]

Host and parasite identity interact in scale-dependent fashion to determine parasite community structure: SUPPLEMENTARY MATERIAL

Joshua I. Brian[1,2], David C. Aldridge[1]

[1]Aquatic Ecology Group, The David Attenborough Building, Department of Zoology, University of Cambridge, Cambridge CB2 3QZ, United Kingdom

[2]Present address: Department of Geography, Bush House North East, King's College London, London WC2B 4BG, United Kingdom

Supplementary Figures

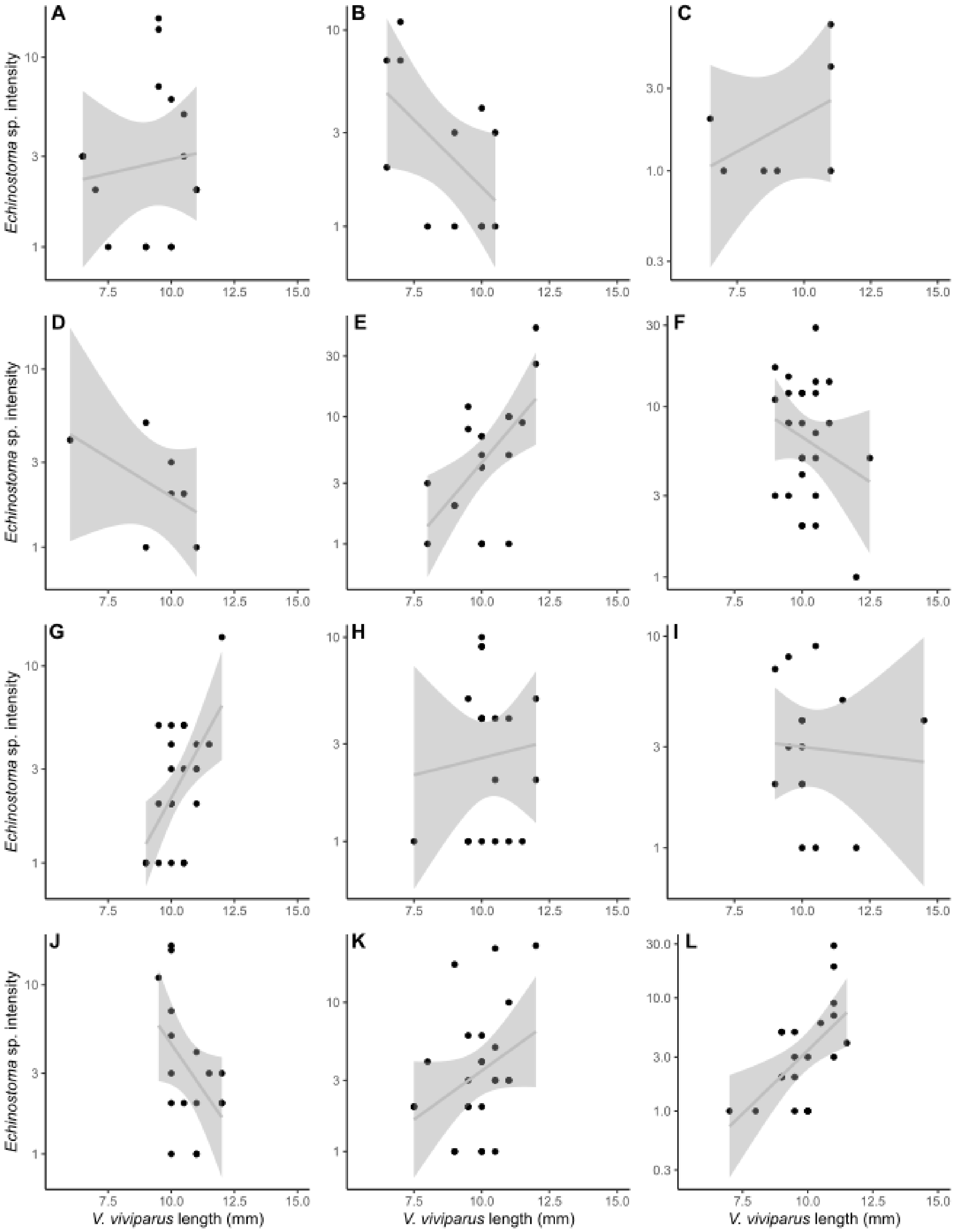

Figure S1: Relationship between *V. viviparus* length and *Echinostoma* sp. intensity for each month from February 2019 to February 2020 (A – L). All x-axes are shown on the same scale, to highlight the observed length:month interaction is due to highly variable intensities and random changes in the minimum and/or maximum host sizes across months. Note that Y-axes are logged.

Supplementary Tables

Table S1: Distribution of host lengths through time. SE = Standard Error.

Sampling date	Host species	Mean length (mm) ± SE
6th March 2019	*A. anatina*	63.77 ± 1.40
3rd April 2019	*A. anatina*	62.21 ± 1.40
7th May 2019	*A. anatina*	62.34 ± 1.29
4th June 2019	*A. anatina*	63.53 ± 1.37
25th June 2019	*A. anatina*	58.38 ± 1.66
12th August 2019	*A. anatina*	60.50 ± 1.40
5th September 2019	*A. anatina*	63.60 ± 1.65
2nd October 2019	*A. anatina*	68.51 ± 1.11
7th November 2019	*A. anatina*	67.41 ± 1.36
2nd December 2019	*A. anatina*	65.31 ± 1.31
20th January 2020	*A. anatina*	64.89 ± 1.26
24th February 2020	*A. anatina*	66.49 ± 1.41
6th March 2019	*V. viviparus*	9.17 ± 0.30
3rd April 2019	*V. viviparus*	8.93 ± 0.24
7th May 2019	*V. viviparus*	8.68 ± 0.24
4th June 2019	*V. viviparus*	9.32 ± 0.29
25th June 2019	*V. viviparus*	10.02 ± 0.18
12th August 2019	*V. viviparus*	10.20 ± 0.15
5th September 2019	*V. viviparus*	10.17 ± 0.21
2nd October 2019	*V. viviparus*	10.00 ± 0.27
7th November 2019	*V. viviparus*	10.30 ± 0.25
2nd December 2019	*V. viviparus*	10.23 ± 0.19
20th January 2020	*V. viviparus*	9.70 ± 0.22
24th February 2020	*V. viviparus*	9.70 ± 0.19

Table S2: Final model summaries for *Echinoparyphium recurvatum*. Reference levels for categorical variables are subsumed into the 'Intercept' term. SE = Standard Error. Individual rows provide information about a deviation from the reference level (host species, month, castrators) or about a one-unit increase in the variable (host length). β-parameters and standard errors have been left in logged form, and can be back-transformed to odds ratios using e^{β}.

(a) Prevalence				
**Variable**	**Level**	**β**	**SE**	**p-value**
Intercept	N/A	0.132	0.609	0.828
Host species	*V. viviparus*	-3.601	0.632	<0.001*
Month	2	0.215	0.365	0.556
	3	-0.956	0.441	0.030*
	4	-0.821	0.440	0.062
	5	-0.968	0.454	0.033*
	6	-0.787	0.429	0.067
	7	-0.955	0.452	0.035*
	8	-0.019	0.383	0.960
	9	0.274	0.378	0.468
	10	-0.492	0.421	0.243
	11	-0.123	0.402	0.761
	12	0.335	0.381	0.380
Castrators†	Yes	0.276	0.246	0.262
Host length	N/A	-0.020	0.009	0.023*
Host species: Castrators	*V. viviparus*: Yes	3.470	0.542	<0.001*
(b) Intensity				
**Variable**	**Level**	**β**	**SE**	**p-value**
Intercept	N/A	-0.416	0.537	0.438
Host species	*V. viviparus*	2.129	0.575	<0.001*
Length	N/A	0.023	0.008	0.005*
Castrators	Yes	-0.538	0.222	0.016*
Host species: Castrators	*V. viviparus*: Yes	0.958	0.441	0.030*

*Significant p-value at $\alpha = 0.05$

†Included in final model due to interaction with other terms only

Table S3: Final model summaries for *Echinostoma* sp. Reference levels for categorical variables are subsumed into the 'Intercept' term. SE = Standard Error. Individual rows provide information about a deviation from the reference level (month, castrators) or about a one-unit increase in the variable (host length). β-parameters and standard errors have been left in logged form, and can be back-transformed to odds ratios using e^{β}.

(a) Prevalence				
**Variable**	**Level**	**β**	**SE**	**p-value**
Intercept	N/A	-3.506	1.237	0.005*
Month	2	-1.762	0.643	0.006*
	3	-1.594	0.718	0.026*
	4	-2.000	0.716	0.005*
	5	-0.045	0.605	0.940
	6	17.05	81.46	0.983
	7	0.603	0.657	0.359
	8	0.525	0.698	0.451
	9	-0.563	0.616	0.360
	10	-0.435	0.602	0.469
	11	1.305	0.671	0.052
	12	0.631	0.616	0.305
Length	N/A	0.397	0.119	0.001*
Castrators†	*V. viviparus*	3.709	2.073	0.074
Length: Castrators	Length: *V. viviparus*	-0.493	0.235	0.036*
Month: Castrators	2: *V. viviparus*	2.150	1.125	0.056
	3: *V. viviparus*	1.655	1.267	0.192
	4: *V. viviparus*	2.610	1.329	0.049*
	5: *V. viviparus*	18.28	19.75	0.993
	6: *V. viviparus*	-15.98	81.46	0.984
	7: *V. viviparus*	17.73	16.14	0.991
	8: *V. viviparus*	0.002	1.255	0.999
	9: *V. viviparus*	1.366	1.405	0.331
	10: *V. viviparus*	2.262	1.565	0.148
	11: *V. viviparus*	17.02	39.56	0.997
	12: *V. viviparus*	0.180	1.824	0.921

(b) Intensity				
**Variable**	**Level**	**β**	**SE**	**p-value**
Intercept	N/A	0.367	1.261	0.771
Month	2	3.721	1.825	0.041*
	3	-0.206	2.345	0.379
	4	2.255	2.231	0.312
	5	-5.198	2.053	0.011*
	6	3.307	2.110	0.117
	7	-5.093	2.741	0.063
	8	0.954	2.264	0.674
	9	1.390	2.047	0.497
	10	8.460	2.944	0.004*
	11	-2.325	1.953	0.234
	12	-5.568	2.216	0.012*
Length†	N/A	0.116	0.134	0.386

Month: Length	2: Length	-0.460	0.206	0.026*
	3: Length	0.153	0.243	0.528
	4: Length	-0.300	0.240	0.211
	5: Length	0.542	0.205	0.008*
	6: Length	-0.269	0.214	0.208
	7: Length	0.447	0.269	0.097
	8: Length	-0.119	0.226	0.598
	9: Length	-0.159	0.205	0.438
	10: Length	-0.810	0.286	0.005*
	11: Length	0.246	0.200	0.221
	12: Length	0.558	0.224	0.013*

*Significant p-value at $\alpha = 0.05$

†Included in final model due to interaction with other terms only
